# Associative Increases in Amyotrophic Lateral Sclerosis Survival Duration With Non-invasive Ventilation Initiation and Usage Protocols

**DOI:** 10.3389/fneur.2018.00578

**Published:** 2018-07-12

**Authors:** Nishad Khamankar, Grant Coan, Barry Weaver, Cassie S. Mitchell

**Affiliations:** ^1^Laboratory for Pathology Dynamics, Biomedical Engineering, Georgia Institute of Technology and Emory University School of Medicine, Atlanta, GA, United States; ^2^School of Medicine, University of Texas Health Science Center at San Antonio, San Antonio, TX, United States

**Keywords:** non-invasive ventilation, palliative care, neuromuscular disease, respiratory intervention, survival duration

## Abstract

**Objective:** It is hypothesized earlier non-invasive (NIV) ventilation benefits Amyotrophic Lateral Sclerosis (ALS) patients. NIV typically consists of the removable bi-level positive airway pressure (Bi-PAP) for adjunctive respiratory support and/or the cough assist intervention for secretion clearance. Historical international standards and current USA insurance standards often delay NIV until percent predicted forced vital capacity (FVC %predict) is <50. We identify the optimal point for Bi-PAP initiation and the synergistic benefit of daily Bi-PAP and cough assist on associative increases in survival duration.

**Methods:** Study population consisted of a retrospective ALS cohort (Emory University, Atlanta, GA, USA). Primary analysis included 474 patients (403 Bi-PAP users, 71 non-users). Survival duration (time elapsed from baseline onset until death) is compared on the basis of Bi-PAP initiation threshold (FVC %predict); daily Bi-PAP usage protocol (hours/day); daily cough assist usage (users or non-users); ALS onset type; ALSFRS-R score; and time elapsed from baseline onset until Bi-PAP initiation, using Kruskal-Wallis one-way analysis of variance and Kaplan Meier.

**Results:** Bi-PAP users' median survival (21.03 months, IQR = 23.97, *N* = 403) is significantly longer (*p* < 0.001) than non-users (13.84 months, IQR = 11.97, *N* = 71). Survival consistently increases (*p* < 0.01) with FVC %predict Bi-PAP initiation threshold: <50% (20.3 months); ≥50% (23.60 months); ≥80% (25.36 months). Bi-PAP usage >8 hours/day (23.20 months) or any daily Bi-PAP usage with cough assist (25.73 months) significantly (*p* < 0.001) extends survival compared to Bi-PAP alone (15.0 months). Cough assist without Bi-PAP has insignificant impact (14.17 months) over no intervention (13.68 months). Except for bulbar onset Bi-PAP users, higher ALSFRS-R total scores at Bi-PAP initiation significantly correlate with higher initiation FVC %predict and longer survival duration. Time elapsed since ALS onset is not a good predictor of when NIV should be initiated.

**Conclusions:** The “optimized” NIV protocol (Bi-PAP initiation while FVC %predict ≥80, Bi-PAP usage >8 h/day, daily cough assist usage) has a 30. 8 month survival median, which is double that of a “standard” NIV protocol (initiation FVC %predict <50, usage >4 h/day, no cough assist). Earlier access to Bi-PAP and cough assist, prior to precipitous respiratory decline, is needed to maximize NIV synergy and associative survival benefit.

## Introduction

Amyotrophic lateral sclerosis (ALS) is a progressive neurodegenerative disorder, characterized by loss of motor neurons (1–4) in the spinal cord and brain (5, 6). The median age of ALS onset is 50–60 years (7–10), and half of ALS patients die within 2–3 years of symptom onset (8, 9, 11). The two most common onset modes are limb onset, characterized by extremity weakness or paralysis, and bulbar onset, characterized by speech difficulty and facial weakness; only a small fraction of patients first present with impaired respiration (1, 11–13). Currently, there exists no cure for ALS (12); and the primary marketed ALS etiology-targeted treatment, Riluzole, only extends survival by 1–4 months (14, 15).

Regardless of onset type, patients eventually lose innervation to diaphragm and intercostal muscles, resulting in impaired respiration. In fact, respiratory complications secondary to progressive muscle atrophy are responsible for the majority of ALS patient deaths (11, 13). Assistive interventions, including non-invasive ventilation like bi-level positive airway pressure (Bi-PAP), are commonly prescribed to ALS patients (11, 13, 16–19). In contrast with invasive measures, non-invasive ventilation does not further inhibit swallowing in patients with mild to moderate dysphagia, where at least partial nutrition by mouth is deemed appropriate (20). Moreover, because Bi-PAP is removable, it interferes less with activities, enabling a higher quality of life without the increased risk of pneumonia, a common drawback of invasive ventilation (21). Nonetheless, the impact of Bi-PAP on ALS survival duration has not been examined in a large study population (16), nor is there broad consensus on Bi-PAP usage protocols.

The standard respiratory metrics of forced vital capacity (FVC) and namely percent predicted FVC (FVC %predict), which considers patient age, gender, and height, are the primary metrics used to assess respiratory function in ALS (1). In fact, a recent study found that over 92% of USA and international clinics still use FVC %predict as their primary metric to determine NIV initiation (22). The current USA standard of care dictates a FVC %predict of 50 (i.e., half of the expected FVC value) as the threshold below which Bi-PAP should be initiated in ALS patients (11); this is largely due to USA medical insurances, including Medicare, which require a FVC %predict <50 in order to cover [or pay] for NIV (22). In contrast, internationally, the NIV initiation threshold is highly variable. Many clinics in Europe and Asia begin NIV much earlier, as shown in a recent study examining differences in international standards of care for ALS NIV (22), but over 20% of international clinics actually begin NIV later, well after functional symptoms commence (23). Irrespective, it has long been hypothesized that starting NIV earlier than the <50 FVC %predict threshold could be associated with additional benefits (24, 25), but there has been a lack of data to illustrate this effect in a large-scale study.

While previous work has shown that NIV intervention prolongs life in ALS patients, existent studies are extremely limited by sample size, including well-known studies [e.g., (11, 24, 26, 27)]. The present study has an included sample size four times greater than previous studies. Such a large cohort enables statistically relevant analysis of the ideal FVC %predict threshold for initiating Bi-PAP, the optimal daily Bi-PAP usage time (hours/day), and the evaluation of the adjunctive usage of cough assist for secretion clearance. In fact, cough assist in particular has been highly litigated, particularly in bulbar ALS populations (27–29) where it could carry a higher risk. The goal is to make recommendations regarding NIV (including Bi-PAP and cough assist) initiation and usage that maximize associative survival benefit.

## Methods

A retrospective analysis was performed of a previously collected de-identified data set (30–32) consisting of 1,585 patients seen at a tertiary ALS specialty clinic (Emory University, Atlanta, GA, USA). Metrics for the present study include: baseline visit date (date of first ALS clinic visit); date of patient-reported first ALS symptom onset; onset type (e.g., bulbar, limb, other); existence of co-morbid respiratory conditions; date of initial Bi-PAP prescription; daily Bi-PAP usage time reported at each visit; measured forced vital capacity (FVC) and percent predicted FVC (%predict) at each visit; date of cough assist prescription; cough assist usage at reported at each visit; recorded date of death. Transcription of the original data from the medical records included a quality control check to insure >99% accuracy (30). Internal Review Board approvals were obtained from Georgia Institute of Technology and Emory University.

### Patient inclusion criteria

Strict data completeness and inclusion criteria were utilized to insure analytical veracity. Only deceased ALS patients with complete clinic and Bi-PAP treatment records for all visits were included. “Non-users” never used Bi-PAP at any point during their disease duration and “users” consistently used Bi-PAP on a daily basis for > 3 months prior to death. Of the 1585 ALS patient charts reviewed (see Table 1), 935 patients had a recorded date of death at the time of study data transcription. Of the 935 deceased patients, 461 were excluded because their Bi-PAP usage did not meet the “consistent” usage definition or because they lacked complete records for all clinic visits. The final cohort consisted of a total of 474 patients comprised of 403 Bi-PAP users and 71 non-users (see Table 2A). Retrospective enrollment began in 1999 and concluded in 2015. None of the included patients in this study were transitioned to invasive ventilation, Trilogy, or supplemental oxygen.

**Table 1:** Overall cohort characteristics.

	***N* = 1,585 Patients**
**Gender**	***N (%)***
Male	945 (59.62)
Female	640 (40.38)
**RACE**	
Caucasian	923 (58.23)
African American	196 (12.37)
Hispanic/Latino	19 (1.20)
Asian	17 (1.07)
Native American	1 (0.06)
Mixed/Other	12 (0.76)
Unspecified	417 (26.31)
**ALS ONSET TYPE**	
Limb	1098 (69.27)
Bulbar	428 (27.00)
Other/unclassifiable	59 (3.72)
**ALS ONSET AGE**	
<55 years	509 (32.11)
55–65 years	474 (29.91)
>65 years	602 (37.98)

**Table 2A:** Comparing Bi-PAP users and non-users as a function of onset type.

**Usage class**	***N* (%)**	**Median survival months, (IQR)**
Bi-PAP Users (all)	403 (85.02)	21.03 (23.97)
Bi-PAP Non-Users (all)	71 (14.98)	13.84 (11.97)
Bi-PAP Users (limb)	252 (53.16)	24.13 (24.47)
Bi-PAP Non-Users (limb)	48 (10.13)	13.5 (11.47)
Bi-PAP Users (bulbar)	139 (29.32)	17.97 (17.93)
Bi-PAP Non-Users (bulbar)	21 (4.43)	14.17 (16.43)

### Bi-PAP prescription criteria

Upright FVC %predict was <50; patient-reported new breathlessness or dyspnea regularly impacting sleep or activity; an in-clinic sleep study revealed depressed respiratory function; a pronounced dip (~20%) in FVC compared to previous clinic visit; the presence of depressed negative inspiratory force (NIF).

Co-morbid respiratory illness (defined below) was not an explicit criterion for Bi-PAP prescription. No distinction was made for this study based on Bi-PAP machine brand name or machine type (e.g., standard Bi-PAP vs. Bi-PAP with Average Volume Assured Pressure Support (AVAPS), the latter which maintains consistent tidal volume).

### Threshold for Bi-PAP initiation

Bi-PAP user group (*N* = 403) was sub-divided based on recorded FVC %predict at the initiation of Bi-PAP prescription. Groups were defined in 10% intervals to ensure adequate sample sizes (Table 2B).

**Table 2B:** Comparing Bi-PAP initiation FVC %predict threshold.

**Bi-PAP initiation FVC %predict**	***N* (%)**	**Median survival months, (IQR)**
<50%	201 (49.90)	20.30 (22.06)
≥50%	202 (50.10)	23.60 (24.40)
≥60%	141 (34.99)	24.10 (21.80)
≥70%	87 (21.59)	24.13 (22.83)
≥80%	44 (10.92)	25.36 (20.40)
≥90%	23 (5.71)	27.70 (27.43)

### Bi-PAP daily usage protocol

Bi-PAP users were divided into the following daily usage time classifications: did not use Bi-PAP; used Bi-PAP <4 h/day; used Bi-PAP ≥ 4 but ≤ 8 h/day, and used Bi-PAP >8 h/day. Analyzed patients had a consistent usage classification constant from Bi-PAP initiation until death (*N* = 210, Table 2C).

**Table 2C:** Comparing Bi-PAP daily usage protocols (hours/day).

**Daily Bi-PAP usage protocol**	***N* (%)**	**Median survival months, (IQR)**
<4 h/day	29 (7.20)	15.07 (22.97)
4–8 h/day	57 (14.14)	21.17 (18.97)
>8 h/day	123 (30.52)	23.20 (29.90)

### Cough assist usage

Cough assist is an intervention that helps with secretion clearance by placing positive pressure and then quickly switching to negative pressure to induce a natural cough. Patients were classified on the basis of whether they consistently used prescribed cough assist on a daily basis (Table 2D), which was defined as at least one cough assist session per day.

**Table 2D:** Comparison of Bi-PAP and cough assist usage.

**Cough assist usage groups**	***N* (%)**	**Median survival months, (IQR)**
Bi-Pap (+), Cough Assist (+)	183 (38.61)	25.73 (21.27)
Bi-PAP (+), Cough Assist (–)	218 (45.99)	15.00 (20.77)
Bi-Pap (–), Cough Assist (+)	17 (3.59)	14.17 (10.73)
Bi-PAP (–), Cough Assist (–)	56 (11.81)	13.68 (13.09)
Bi-PAP (±), Cough Assist (+)	200 (42.19)	24.38 (22.32)
Bi-PAP (±), Cough Assist (–)	274 (57.81)	14.87 (18.53)
Bi-PAP (+), Cough Assist (±)	403 (85.02)	21.03 (23.97)

### Survival duration calculation

Survival duration was calculated and compared using two different definitions: (1) time elapsed from the patient's first or “baseline” tertiary ALS clinic visit until death—a definition that has proven to be most reliable for clinical analysis (33, 34); and (2) time elapsed from the patient's first reported symptom or “true onset” until death, a definition preferred for its ease of intuitive understanding but confounded by patient recall bias or lack of normalization (33, 34). Unless otherwise noted, survival durations (in months) are presented as a median with interquartile range (IQR). While calculations were performed and compared using both definitions of survival onset, the first or “baseline” definition is used for consistency within the text and presented tables and figures, except where otherwise noted.

### Temporal comparisons and disease quartiles

In order to better assess how time elapsed since disease start (using both the “true onset” and “baseline” definitions) until Bi-PAP initiation could be associated with survival benefit, the time(s) from from true onset and baseline until Bi-PAP initiation was calculated and compared between different Bi-PAP user groups. Disease quartile comparisons, where a quartile represents each 25% increment from true onset or baseline until death, were calculated. The first quartile represents the first 25%, the second quartile 26–50%, the third quartile 51–75%, and the fourth quartile 76–100% of time elapsed [since true onset or baseline] until death. Bi-PAP initiation within each quartile was compared to determine if time since onset or baseline is a predictor of associative survival benefit.

### Antecedent or co-morbid respiratory disease

Patients with confirmed antecedent or co-morbid respiratory conditions, such as COPD, lung cancer, and severe asthma were identified using previously published protocols (31, 32) and separately compared to ALS patients without such disease to identify any possible result-influencing confounds.

### ALS onset type

ALS patients were classified as either “limb onset,” “bulbar onset,” or “other/unclassifiable” based on reported first symptoms according to standard published definitions (35). Patients with recorded mixed initial onset symptoms or those that did not clearly or definitively meet the limb or bulbar definition were classified as “other/unclassifiable.”

### Statistical analysis

The distribution type was found to be non-normal using a Shapiro-Wilks test. Thus, median survival durations were compared via a Kruskal-Wallis one-way analysis of variance with a significance *p*-value threshold of 0.05. Additionally, a Kaplan Meier analysis was used to assess probability of survival over time. The present Kaplan Meier plots to visualize survival probability trends from baseline (0 months) to 60 months, a time period where survival differences and samples sizes are largest. Note that the sample sizes of surviving patients beyond 60 months is small.

## Results

### Cohort characteristics

Overall selection cohort (*N* = 1,585, Table 1) characteristics are similar to literature-cited ratios for gender, ethnicity, onset type, and onset age (5, 8, 19, 35). Based on inclusion criteria (see section Methods), 403 Bi-PAP users and 71 non-users were included for analysis. The onset type and onset age distributions of the included patients (*N* = 474) were similar to the overall cohort. Bi-PAP users are further classified by FVC %predict value at Bi-PAP initiation (*N* = 403) and consistent daily Bi-PAP usage time classification (hours/day) from Bi-PAP initiation until death (*N* = 210). Sub-analyses (*N* = 474) were also performed to explicitly examine Bi-PAP users and Bi-PAP non-users on the basis of whether they used cough assist.

Both the “true onset” and “baseline” onset definitions (see section Methods) were initially used to calculate survival duration and other temporal metrics of disease progression. There was no statistical difference in the two definitions when comparing measured differences between sub-populations. Because of the indistinguishable difference on calculated statistical results, the “baseline” definition is used in the presented results and figures given its prior determination as the preferred literature standard for comparing disease progression (33, 34, 36).

### Bi-PAP users vs. non-users by onset type

Table 2A illustrates the breakdown of major classes of Bi-PAP users strictly on the basis of their using Bi-PAP consistently from the time Bi-PAP was initiated until death. The median survival duration for all Bi-PAP users (*N* = 403) was found to be 21.03 months (IQR = 23.97 months), while all non-users was 13.84 months (IQR = 11.97 months). The Bi-PAP users survived significantly longer than non-users (*p* << 0.001), resulting in an average associative survival benefit of 8.19 months, a 52% increase in survival duration. Limb onset Bi-PAP users have a median survival of 24.13 months and bulbar onset Bi-PAP users 17.97 months compared to 13.5 and 14.17 months for limb and bulbar onset non-users, respectively. Thus, the limb onset Bi-PAP users had a 79% associative increase in survival duration whereas bulbar onset Bi-PAP had a 26.8% associative increase in survival duration. 12 Bi-PAP users and 2 Bi-PAP non-users were unable to be classified by onset type (see section Methods).

### Forced vital capacity threshold for Bi-PAP initiation

Table 2B compares survival duration among Bi-PAP users on the basis of percent predicted forced vital capacity (FVC %predict) at Bi-PAP initiation. Historically in the non-invasive ventilation ALS literature (11, 22), and presently for the sake of Bi-PAP financial coverage by private and/or government medical insurance in the United States, an FVC %predict < 50 is employed as the standard threshold value to initiate Bi-PAP intervention in ALS. The FVC %predict is calculated using expected FVC values for a given patient age, gender, and height (1). A FVC %predict of 50 equates to respiratory function that is only half of the expected value in an equivalent non-diseased patient. An examination of the 50% threshold in ALS Bi-PAP users reveals a significant difference in survival duration between patients initiating Bi-PAP below the FVC %predict threshold of 50 (*N* = 201, median = 20.30 months) and those at or above the 50 %predict threshold (*N* = 202, median = 24.10 months) at the time of Bi-PAP initiation, with the latter group having a significant 18.7% associative increase in survival duration (*p* < 0.01).

Analyses using higher Bi-PAP initiation FVC %predict threshold values (60, 70, 80, and 90) were explored to determine if earlier Bi-PAP initiation is associated with longer survival duration (Table 2B). Increasing the FVC %predict threshold to ≥ 60 (*N* = 250, median = 24.10 months) resulted in a significant 18.7% increase in survival duration compared to the standard < 50 FVC %predict threshold (*p* < 0.001). The ≥ 70 FVC %predict group was nearly identical to the ≥ 60 group. However, the ≥ 80 %predict Bi-PAP initiation group (*N* = 44) has a significant 25% associative increase in survival duration (p < 0.01) over the standard < 50 FVC %predict threshold group. Those with FVC %predict ≥90 at Bi-PAP initiation (*N* = 23) lived an astounding 36.5% longer (*p* < 0.01) than users in the standard threshold (FVC %predict < 50) group.

### Assessment of daily Bi-PAP usage protocol

Table 2C compares the daily usage protocols of Bi-PAP users, which includes classes of users that remained on the same daily usage protocol from Bi-PAP initiation until death. 210 of the 403 total Bi-PAP users were included in daily usage protocol analysis due the stipulation that users remain in the same usage protocol classification (hours/day) from Bi-PAP initiation until death. A Kruskal-Wallis comparison of survival duration between patients who consistently used Bi-PAP < 4 h/day (*N* = 30), 4–8 h/day (*N* = 57), and > 8 h/day (*N* = 123) was performed. Statistically significant differences in survival duration between the daily usage groups were only found between the < 4 h/day and > 8 h/day (*p* < 0.05). Overall, these results suggest that while associative survival benefit is present across every Bi-PAP daily usage protocol, maximal associative survival benefit requires > 8 h/day of Bi-PAP usage.

### Comparing Bi-PAP and cough assist

Table 2D compares the impact of cough assist usage among Bi-PAP users and non-users. Irrespective of Bi-PAP usage, all cough assist users [cough assist (+), Bi-PAP (±); median = 24.38 months] lived significantly longer (*p* < 0.0001) than all patients that did not use cough assist [cough assist (–), Bi-PAP (±); median = 14.87 months]. Among patients that consistently used both Bi-PAP and cough assist [Bi-PAP (+), Cough Assist (+); median = 25.73 months], there is a significant 88% associative increase (*p* < 0.0001) over those that used neither intervention [Bi-PAP (–), Cough Assist (–); median = 13.68 months]. Interestingly, there is a significant difference (*p* < < 0.001) between Bi-PAP users who also used cough assist [Bi-PAP (+), cough assist (+); median = 25.73 months] compared to Bi-PAP users who did not use cough assist [Bi-PAP (+), cough assist (–); median = 15.0 months]. However, there was no significant difference (*p* > 0.05) between Bi-PAP non-users who used cough assist [Bi-PAP (–), cough assist (+); median = 14.17 months] vs. those who used neither intervention [Bi-PAP (–), cough assist (–); median = 13.68 months].

### Comparing ALSFRS-R score and time elapsed since Bi-PAP initiation

Table 3 illustrates the median revised Amyotrophic Lateral Sclerosis Functional Rating Scale (ALSFRS-R) score at Bi-PAP initiation and the time elapsed (in months) from baseline until Bi-PAP initiation. The ALSFRS-R (33) is series of 12 survey questions with a degree of impairment scale ranging from (4 = normal) to (0 = unable to perform task). The questions predominantly cover activities of daily living that take into account skeletal muscle function, respiratory function, and swallowing ability, where a “normal” total score in a person with no impairment equates to 48 (e.g., 12 x 4 = 48). Bulbar onset Bi-PAP users have a median ALSFRS-R score of 31 at the time of Bi-PAP initiation compared to limb onset Bi-PAP users, which have a median ALSFRS-R score of 25. Bi-PAP bulbar onset patients started Bi-PAP much earlier than limb patients. Time from ALS baseline to Bi-PAP initiation in bulbar onset Bi-PAP users is 5.4 months compared to 10.77 months in limb onset Bi-PAP users. Bi-PAP users in the longer Bi-PAP daily usage (hours/day) categories tend to start Bi-PAP later from ALS onset, although there is no significant difference in daily usage group ALSFRS-R scores at Bi-PAP initiation. Not surprisingly, those patients with better FVC %predict thresholds tend to begin Bi-PAP sooner and have higher ALSFRS-R scores at Bi-PAP initiation compared to patients with lower FVC %predict thresholds at Bi-PAP initiation. There were significant differences (*p* < 0.001) in ALSFRS-R scores between all FVC %predict Bi-PAP initiation threshold sub-groups. There was no significant difference in total ALSFRS-R score at Bi-PAP initiation based on whether Bi-PAP users did or did not use cough assist.

**Table 3:** Comparing ALSFRS-R score and time from onset until Bi-PAP intiation.

**Bi-PAP user sub-group**	**Median ALSFRS-R Score, (IQR)**	**Median time months, (IQR)**
Bulbar onset	31 (13)	5.40 (9.79)
Limb onset	25 (12)	10.77 (15.73)
<4 h/day	26 (12)	5.47 (7.89)
4–8 h/day	27 (14)	7.57 (10.25)
>8 h/day	27 (14)	8.68 (12.93)
FVC %predict < 50	22 (13)	9.90 (17.27)
FVC %predict ≥ 50	29 (10)	7.23 (12.34)
FVC %predict ≥ 60	31 (11)	7 (12.29)
FVC %predict ≥ 70	32 (11)	5.57 (10.99)
FVC %predict ≥ 80	34 (13)	5.57 (10.33)

In addition to examining time elapsed from baseline until Bi-PAP initiation, we also examined how normalized disease duration quartiles may be associated with Bi-PAP sub-population survival duration. Interestingly, there was no associative difference in survival duration as a function of what disease quartile the patient was in when Bi-PAP was initiated. That is, there was no significant difference (*p* >> 0.05) in survival duration simply based on starting Bi-PAP in the first, second, third, or fourth quartile of the patient's overall disease duration. Thus, neither time since true onset nor baseline onset is a good predictor of when Bi-PAP should be started or a predictor of its overall associative survival benefit. The lack of a correlation with temporal metrics is likely explained by the highly heterogeneous disease courses among patients.

### Comparing Bi-PAP protocol parameter combinations

The results discussed above individually evaluated the impact of Bi-PAP protocol parameters (e.g., Bi-PAP FVC %predict threshold, Bi-PAP daily usage threshold (hours/day), and concurrent use of cough assist). Additionally, key combinations of relevant parameters were also assessed based on significance identified upon evaluation of the individual parameters. For the combination assessment, the FVC %predict thresholds included > 80, > 60, and < 50; the daily usage (hours/day) included > 8 and > 0 h/day (e.g., < 4 OR 4–8 h/day); and whether cough assist was used [cough assist (+)] or not [cough assist (–)]. The results, including the sample size of patients using each combination (N), the medians ALSFRS-R score at Bi-PAP initiation, and the medians survival (months) is illustrated for each combination in Table 4. The concurrent consistent usage of cough assist has a significant impact irrespective of the other Bi-PAP parameters. In the absence of cough assist [cough assist (–)], the significant differences in median survival durations among the Bi-PAP protocol parameters of initiation FVC %predict and daily usage (hours/day) become even more pronounced. Moreover, there are significant (*p* < 0.001) differences between all > 60 FVC %predict combinations as well as all < 50 FVC %predict combinations. Significant differences in Bi-PAP initiation ALSFRS-R score correlated with the FVC %predict at Bi-PAP initiation; additionally there were significant differences in ALSFRS-R score for all three of the sub-groups who began Bi-PAP at the ≥ 80 FVC %predict threshold.

**Table 4:** Comparing Bi-PAP usage protocol parameter combinations.

**Bi-PAP user sub-group**	***N***	**Median ALSFRS-R at Bi-PAP initiation Score, (IQR)**	**Median survival months, (IQR)**
≥80 %predict, >8 h/day, cough assist (+)	6	37 (3)	30.8 (22.38)
≥80 %predict, >0 h/day, cough assist (+)	22	37 (12)	24.17 (19.50)
≥80 %predict, >0 h/day, cough assist (–)	30	31 (10)	21.12 (22.46)
≥60 %predict, >8 h/day, cough assist (+)	26	33 (11)	25.85 (32.78)
≥60 %predict, >8 h/day, cough assist (+)	72	33 (10)	25.55 (22.92)
≥60 %predict, >0 h/day, cough assist (–)	69	29 (10)	19.53 (23.50)
< 50 %predict, >8 h/day, cough assist (+)	22	20 (8)	29.77 (17.20)
< 50 %predict, >0 h/day, cough assist (+)	73	25 (10)	26.03 (15.20)
< 50 %predict, >0 h/day, cough assist (–)	116	19 (13)	14.03 (18.34)

Based on prior literature (28, 37), we also examined the bulbar patients separately as cough assist was previously questioned as perhaps “not a good idea” in bulbar ALS patients, largely due to potential laryngeal issues. However, in this study cohort, bulbar patients did have an increase in survival duration with cough assist. Bulbar patients who only used cough assist [bulbar, Bi-PAP (−), cough assist (+)] had a median survival duration of 18.14 months, bulbar patients who used both Bi-PAP and cough assist [bulbar, Bi-PAP (+), cough assist (+)] had a median survival duration of 18.6 months, and bulbar patients that had no intervention had a median survival duration of 9.43 months [bulbar, Bi-PAP (−), cough assist (−)]. Thus, cough assist significantly associated with a positive increase in survival duration over no intervention at all. However, the synergistic gains of using Bi-PAP and cough assist in combination were not nearly as pronounced in the bulbar onset group as the limb onset group.

**Figure 1:**
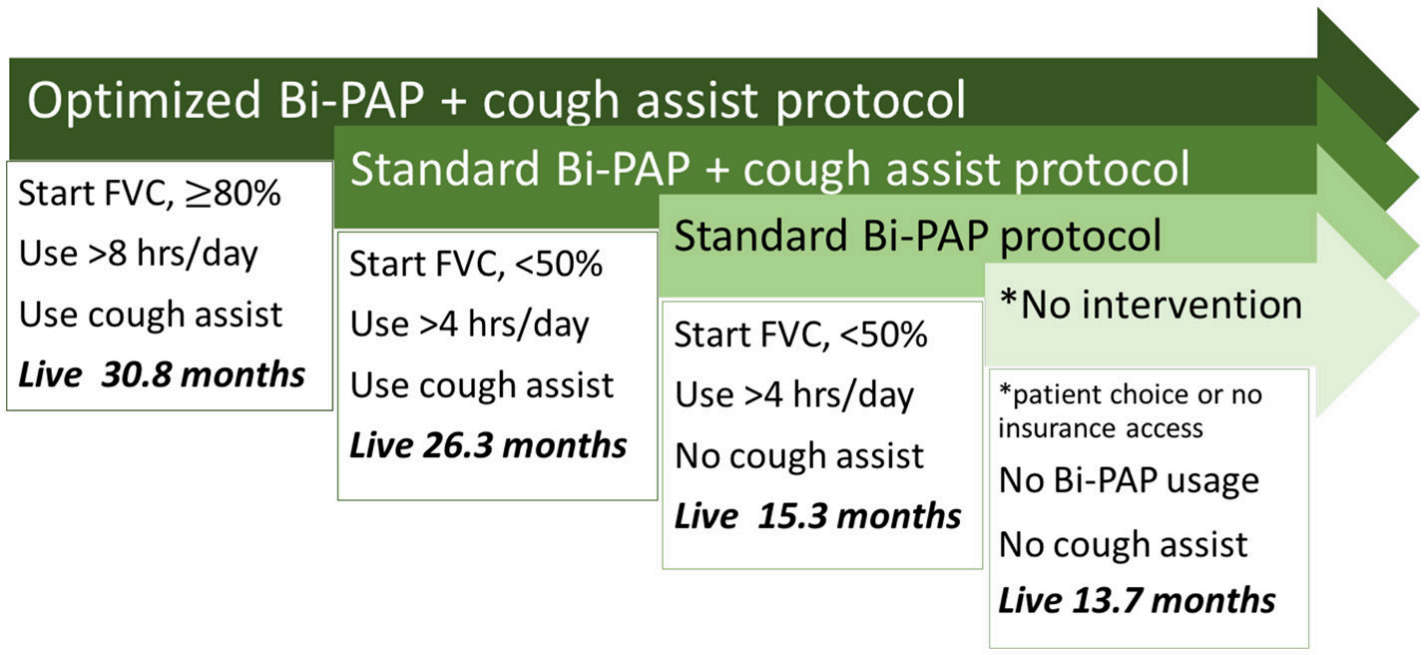
Overview of associative survival duration differences between NIV protocols. There are significant differences in survival duartion among each of the illustrated protocols.

Figure 1 summarizes and compares the significant difference in median survival durations among the optimized Bi-PAP usage + cough assist protocol, standard Bi-PAP + cough assist protocol, standard Bi-PAP without cough assist protocol, and the no intervention protocols. Median survival durations range from 30.8 months (optimized Bi-PAP with cough assist) to just 13.7 months (no intervention).

### Comparing temporal survival probability using kaplan meier

**Figure 2:**
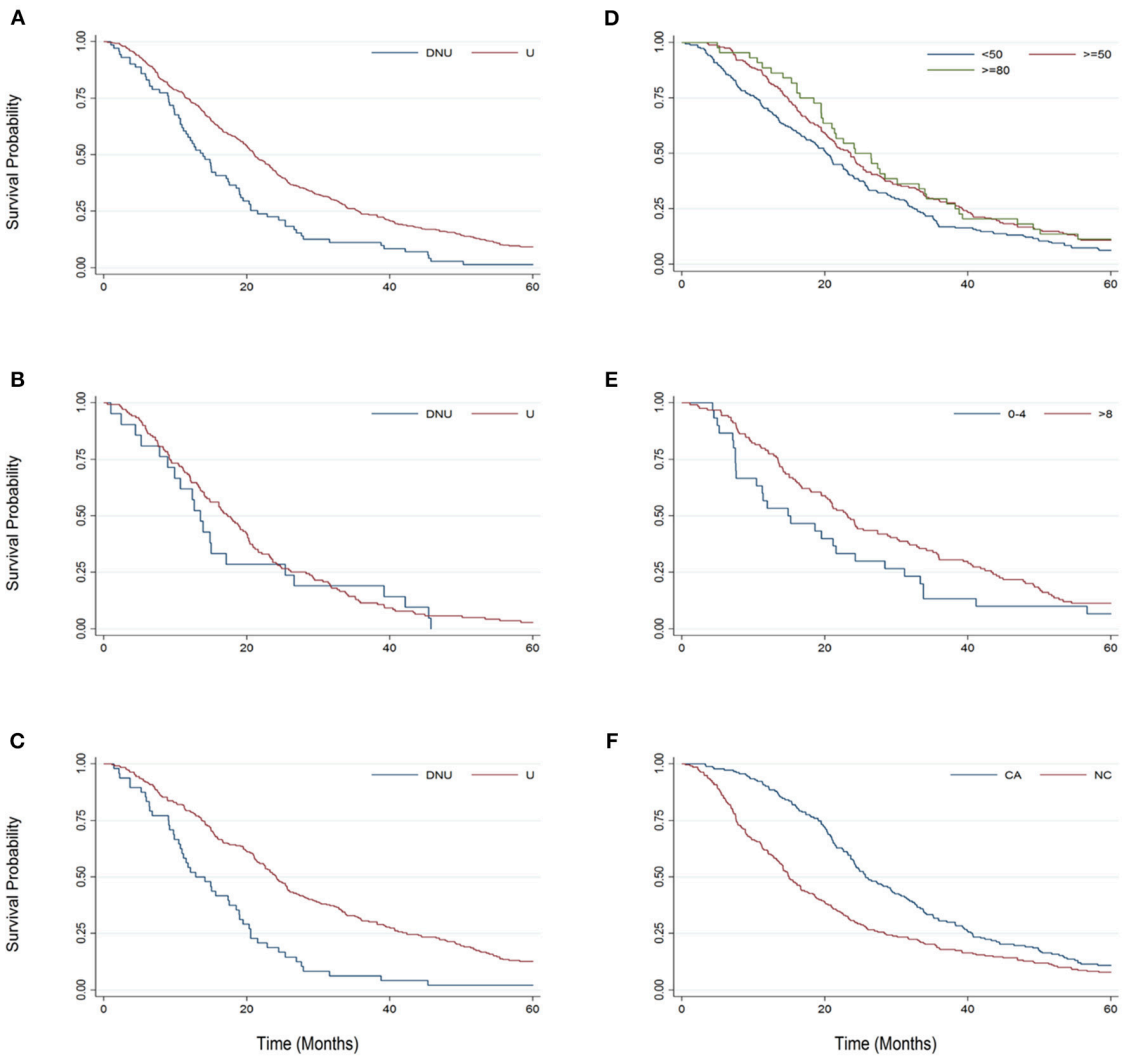
Kaplan-Meier graphs comparing survival probability from 0 to 60 months from baseline for key sub-group pairings. **(A)** Bi-PAP users (U) and non-users (DNU). **(B)** Bulbar onset Bi-PAP users (U) and non-users (DNU). **(C)** Limb onset Bi-PAP users (U) and non-users (DNU). **(D)** BiPAP users classified by the FVC %predict at which they initiated Bi-PAP: <50, ≥50, and ≥80. **(E)** Bi-PAP users classified by their Bi-PAP daily usage time: <4 h/day and ≥ 8 h/day. **(F)** BiPAP users who also used cough assist (CA) or never used cough assist (NC).

Examining median changes, such as median survival, gives a straightforward and meaningful metric to compare different protocols. However, the median, alone, does not always present the whole picture, especially in very heterogeneous populations where the variance, particularly in survival duration, is high. Kaplan Meier analysis is a statistical method that examines survival probability over time. Figure 2 examines survival probability curves generated from Kaplan Meier analysis for key pairings for the first 60-months (5-years) from baseline. With the exception of Figure 2F, each Bi-PAP cohort sub-grouping includes both cough assist users and non-users [e.g., cough assist (±)]. All Bi-PAP users have a higher survival probability than Bi-PAP non-users throughout the 60-months from baseline (Figure 2A). The difference in survival among Bi-PAP users and non-users is more stark in limb onset patients (Figure 2C) than bulbar onset patients (Figure 2B). Bi-PAP initiation FVC %predict threshold (Figure 2D) shows a clear trend of associated prolonged increase in survival with higher FVC %predict thresholds, although differences in survival probability are most pronounced between 12 to 36 months from baseline. The difference in using Bi-PAP <4 h/day and >8 h/day are quite stark throughout (Figure 2E). Finally, the difference in survival probability among Bi-PAP users who also used cough assist was greatly improved throughout compared to Bi-PAP users who did not use cough assist (Figure 2F).

**Figure 3:**
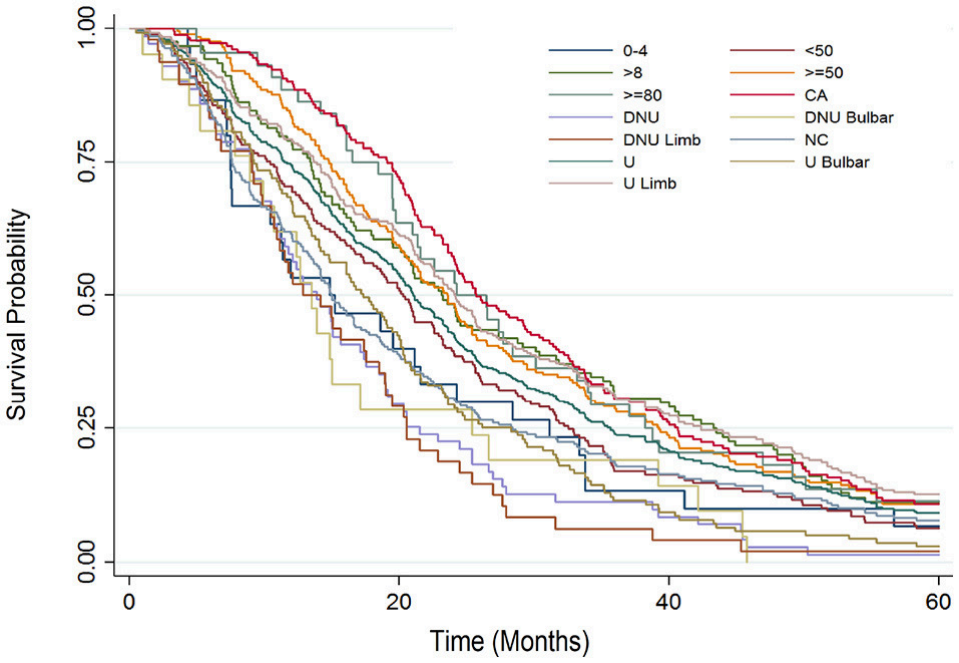
Kaplan Meier survival analysis summary examining surival probability from 0 to 60 months from baseline for each major study sub-group: all Bi-PAP users (U), all Bi-PAP non-users (DNU), all limb onset Bi-PAP users (U limb), all bulbar onset Bi-PAP users (U bulbar), all limb onset Bi-PAP non-users (DNU limb), all bulbar onset Bi-PAP non-users (DNU bulbar), all Bi-PAP users who also used cough assist (CA), all Bi-PAP users who never used cough assist (NC), all Bi-PAP users with <4 h/day of usage, all Bi-PAP users with >8 h/day of usage, all Bi-PAP users who initiated Bi-PAP with a FVC %predict <50 (<50), all Bi-PAP users who initiated Bi-PAP with a FVC %predict ≥50 (≥50), all Bi-PAP users who initiated Bi-PAP with a FVC %predict ≥80 (≥80).

Figure 3 presents a Kaplan Meier survival analysis summary for each individual major sub-group protocol parameter. Again, survival probability is compared from 0 to 60 months from baseline. The two sub-groups that fared comparatively the best were the Bi-PAP users that also used cough assist and the Bi-PAP users who initiated Bi-PAP at an FVC %predict ≥80.

### Comparing benefits of Bi-PAP and riluzole

For the sake of comparison, we calculated the associative survival benefit of riluzole, the first prescribed ALS-specific treatment, for all included patients with a known date of death. Note that because of the retrospective enrollment end date of this study, the newer ALS drug, edaravone, had not yet been FDA approved for ALS for this United States study population. [Edaravone was not FDA approved in the USA until 2017.]. Thus, comparison data for this cohort was only available for riluzole.

Just under 60% of the 935 patients with a recorded date of death used riluzole at some point during their disease, resulting in a +1.5-month associative increase in survival duration. A +2.4-month associative increase in duration was seen for the 20% of patients who took riluzole throughout their disease course. These associative riluzole survival benefits are similar to previous studies [14, 15]. In contrast, the overall associative survival benefit was +7.4-months for all Bi-PAP users regardless of protocol parameters and +17.1-month for Bi-PAP users on the “optimized” Bi-PAP protocol (started Bi-PAP while FVC %predict ≥ 80, Bi-PAP daily usage > 8h/day, used cough assist). Of course, the riluzole and Bi-PAP cohorts have overlap in that about half of the Bi-PAP patients took riluzole at some point during their disease. Nonetheless, the comparison highlights the additional value of Bi-PAP and cough assist.

### Assessment of possible confounds

We separately analyzed patients with known antecedent/or co-morbid respiratory illness. Interestingly, the respiratory illness group exhibited a slightly longer, albeit statistically insignificant (*p* >> 0.05), increase in survival duration, which is consistent with previous work (31, 32). Since no significant difference was detected, Bi-PAP usage analyses did not differentiate patients on the basis of antecedent and/or comorbid respiratory illness. Co-morbid or antecedent respiratory patients made up an insignificantly larger percent of Bi-PAP users (15%) compared to non-users (13%).

ALS patients with limb onset and/or a younger onset age tend to live longer, an assertion strongly supported in the literature (35). The impact of onset type in Bi-PAP usage has already been examined in Table 2A, Figures 2, 3 and in “Comparing Bi-PAP protocol parameter combinations”; these examinations illustrate that, regardless of NIV protocol, bulbar patients do have lesser survival duration although Bi-PAP and/or cough assist usage nonetheless is still associated with a significant increase in survival.

Overall, there was no significant difference in ALS onset age distribution between Bi-PAP users vs. non-users. However, the age distribution of the bulbar Bi-PAP users was significantly older (*p* < 0.01) with 19.42% having an onset age of <55 years, 39.57% with an onset of 55–65 years, and 40.29% having an onset age > 65 years. A previous study has hypothesized that NIV benefit is a function of patient age (38), with older patients benefitting more. However, in the present cohort, there was not a clear correlation of associative benefit solely as a function of patient age.

For additional confounding factors not assessed, please see the *Limitations* sub-section in the Discussion.

## Discussion

Our results demonstrate NIV usage, including Bi-PAP and/or cough assist, is associated with significant increases in survival duration. The present study has the advantage of a large sample size (403 Bi-PAP users, 71 non-users, total *N* = 474) compared to previous similar studies, such as that conducted by Kleopa et. al. (70 NIV users, 52 non-users, total *N* = 122) (11), the studies by Bourke et al. (26, 39), which had an enrollment of 15 and 92 subjects, respectively. All of the other NIV studies also had samples sizes of <100 [e.g.,(16, 28, 29, 40)]. The large sample size of the present study provides confidence in the associative survival benefit of NIV. Irrespective of initiation threshold or hours/day usage protocol, Bi-PAP users lived 7.35 months longer, and patients that used both Bi-PAP and cough assist lived 12.05 months longer. In fact, the associative survival benefit of NIV in the present cohort, was 3 to 7 times larger than that of the ALS-specific drug, riluzole. The degree of associative benefit varied as a function of Bi-PAP initiation threshold, hours/day of Bi-PAP usage, and the daily usage of cough assist (Figure 1). Notably, we saw that even bulbar patients, where NIV has been more controversial, had significant increases in survival. Our results support another recent study (29), which also found that bulbar patients benefitted significantly from NIV despite the greater risks with bulbar dysfunction.

Analysis of the FVC %predict threshold reveals that associative survival benefit increases when patients begin Bi-PAP prior to precipitous respiratory decline. Historical literature and current USA medical insurance standards recommend Bi-PAP usage be prescribed to patients only once their FVC %predict falls below 50%, unless a precipitous decline is noted or dyspnea is observed (11, 41). However, other international ALS clinics have certainly promoted earlier non-invasive for several years based on their own clinical observations, which supported earlier non-invasive ventilation paradigms like Bi-PAP (42). The presented analyses showed significant (*p* < 0.01) increases in survival duration for those starting ≥ 60, ≥ 70, ≥ 80, or ≥ 90 FVC %predict when compared to those starting at ≤ 50 FVC %predict. Based on the statistically definitive results of this analysis, we assert that the FVC %predict threshold value for Bi-PAP treatment initiation should be no less than 80%. Moving the Bi-PAP initiation threshold to ≥ 80 FVC %predict results in an associative 25% longer survival duration than the historical standard of < 50 FVC %predict threshold. The sharp increase in survival duration in the ≥ 90 FVC %predict group, a 36.5% longer survival than the standard 50 FVC %predict threshold, warrants further follow-up with a larger sample size.

There will always be discourse on the validity and accuracy of FVC %predict equations, irrespective of parameters used for the predicted calculation. For example, it has been found that some “normal” or non-pathological patients may have a standard FVC %predict that is ± 20% of the predicted [or expected] value with the standard FVC %predict equation (43). Nonetheless, the present study's analysis clearly shows that earlier intervention is associated with longer survival duration in ALS. Thus, even considering a possible ± 20% range on FVC %predict, changing the threshold for Bi-PAP initiation to ≥ 80% of the predicted value is reasonable. Interestingly, negative inspiratory force (NIF) [also known as maximal inspiratory pressure (MIP)] has previously been analyzed being possibly a better metric for determining NIV initiation as it picks up changes earlier, and enables earlier initiation of Bi-PAP in USA clinics where patients may not yet meet the < 50 FVC %predict threshold required for medical insurance to pay for Bi-PAP (44). As noted in the Methods, the adjunctive use of NIF was indeed one alternative way in which patients in the present study's cohort were able to acquire earlier access to Bi-PAP in terms of USA medical insurance coverage.

It is not clear as to why starting Bi-PAP earlier has such a dramatic effect. This study, alone, cannot distinguish between a causal effect vs. an associative effect of optimized NIV protocols with survival duration. One reason for what we refer to as an “associative increase in survival duration” could be a single FVC reading > 50% in the clinic is not indicative of the stress ALS puts on the system, especially during sleep. In fact, all the patients prescribed Bi-PAP with FVC %predict > 50 reported respiratory symptoms or had measurably impaired respiration by adjunctive metrics. Interestingly, only a handful of study patients had a sleep apnea diagnosis prior to their ALS diagnosis. Another possibility is that early Bi-PAP initiation could be prolonging respiratory innervation by insuring adequate oxygenation and taking some stress off of weakened respiratory muscles. Better respiration could also increase quality of life and will to live. Notably, survival was also strongly tied to ALSFRS-R score at Bi-PAP initiation with those with higher scores surviving longer. Additional studies are needed to better ascertain why optimized Bi-PAP protocols, which are typically considered as palliative only, are associated with such stark increases in survival duration. Interestingly, while we did see significant associative increases in survival duration, the use of NIV did not change the slope of respiratory disease decline (data not shown), which was also highlighted in a recent smaller NIV study (40).

While significant associative survival benefit was present across all Bi-PAP daily usage protocol treatment groups (< 4 h/day, 4–8 h/day, > 8 h/day), significant differences between usage protocols was only present between the < 4 h/day and > 8 h/day usage groups. These results compare favorably to the smaller Kleopa et al study (11). The present study supports a standard protocol of > 8 h/day of Bi-PAP usage, which typically translates to using Bi-PAP overnight or during times of sleep. Although it should be noted that the present study does not discriminate on daily or nightly usage but rather total usage in a 24-h period. Bi-PAP usage while sleeping assists in the additional respiratory challenges when laying horizontal and minimizes interference during wakeful activity.

The concurrent daily usage of cough assist (Table 2D, Figures 1–3) with Bi-PAP has a significant, associative increase in survival duration, especially in limb onset users. The associative impact of isolated cough assist (without Bi-PAP) is not as profound in limb patients, although the impact of isolated cough assist was more profound in bulbar patients. In all patients, the combination of Bi-PAP and cough assist resulted in an associative increase in survival duration. Thus, there appears to be a highly synergistic effect in clearing secretions with daily cough assist usage combined with daily Bi-PAP usage to assist in respiration. The difference is seen not only in the median survival duration (Table 2D), but also in the temporal survival probability as illustrated in Kaplan Meier (Figure 2, 3).

Examining combinations of different Bi-PAP usage protocols reveals that initiation threshold of FVC %predict, daily usage (hours/day), and cough assist usage are all important parameters (Table 4). However, as FVC %predict drops, the impact of the other parameters become even more pronounced. While all parameters are important, the combination of median survival duration and temporal Kaplan Meier survival probability suggests that beyond simply consistently using Bi-PAP each day, the order of protocol parameter importance appears to be: cough assist usage, initiating Bi-PAP earlier when FVC %predict is higher (preferably >80), and using Bi-PAP for >8 h/day. The lack of a significant difference in survival duration and Bi-PAP initiation using temporal disease metrics (e.g., time elapsed since onset to determine Bi-PAP initiation) is interesting. In contrast, time since onset is a strong predictor of survival in population-level machine learning prediction and classification models of ALS survival (36).

### Limitations and future work

Notably, the present study's examination of daily Bi-PAP usage time stipulated that each included patient utilize the same daily usage time protocol from Bi-PAP initiation until death, a criterion that ultimately sacrificed sample size to insure precise categorical comparison. In standard practice, many patients may fall into more than one usage category, as an individual's optimal Bi-PAP usage time is often determined through a trial-and-error process, or Bi-PAP duration is increased as ALS respiratory dysfunction progresses (45). Future work, such as informatics-based analyses (46, 47), is necessary to determine what objective clinical criteria should be used to dynamically determine Bi-PAP usage time as a function of disease stage and patient profile.

FVC was not the only criteria used to prescribe Bi-PAP (see section Methods). However, for many USA medical insurance companies including government-based Medicare, FVC is the primary determining factor for coverage of Bi-PAP. In this USA population, several patients with FVC %predict >50 petitioned insurances via clinician-assisted prior authorizations based on reported symptoms; other respiratory metrics (like NIF) showing impaired function; or sleep studies illustrating respiratory impairment. However, it is possible that personal financial or insurance limitations prevented Bi-PAP access in some cases as not all insurances grant exceptions. A portion of uninsured or denied USA patients are able to personally pay for the Bi-PAP intervention. With the collected data, it was not possible to determine why patients declined to obtain and/or use Bi-PAP even if they met the Bi-PAP prescription criteria stated in the Methods. Other contributors to Bi-PAP patient compliance or refusal of the intervention could be related to depression, will to live, or perceived Bi-PAP side effects (e.g., mask claustrophobia or sleep interference), etc.

Other limitations to the study include the number and types of parameters assessed. In particular, additional assessments of respiratory function could be helpful to find the best metric or combination of metrics for determining NIV initiation. For example, a recent small retrospective study (*N* = 87) by Tilanus et al. found that NIF (also called MIP) and sniff inspiratory nasal pressure (SNIP) were better at identifying the need for earlier NIV (48). Another hotly debated topic is whether FVC %predict should be taken while upright or while laying (22), which should be investigated in greater detail; most USA clinics use the upright metric. Also, additional functional disease progression (e.g., ALSQ40, ALSQ10, bulbar scores, etc.) may be more sensitive than ALSFRS-R, although the ALSFRS-R is still the most widely utilized functional metric. The examination of how NIV impacts quality of life is also important; thus, other quality of life metrics (McGill quality of life scale, neurology quality of life measurement system, etc.) may provide additional insight. Collectively, all of the aforementioned metrics could shed additional light on potential causal or associative reasons why earlier NIV initiation has a significant associative correlation with increased survival duration.

Future work includes not only dynamic NIV assessment with disease progression, but also optimization of specific NIV machine settings, specific Bi-PAP or cough assist machine types, and combined assessment with newer interventional ALS medications like edaravone.

## Conclusions

In summary, as shown in Figure 1, we propose that clinician prescriber and/or medical insurance carriers facilitate earlier NIV intervention. In particular, Bi-PAP should be initiated while ALS patients have a recorded FVC %predict ≥ 80; Bi-PAP should be used at least 8 h/day; and cough assist should be used daily to assist with secretion clearance. The aforementioned “optimized” NIV protocol can extend life by a factor of up to 2 compared to the standard Bi-PAP protocol and a factor of 2.25 compared to no intervention. The overall conferred survival benefit of optimized NIV protocol is even more impressive than the ALS drug, riluzole. Finally, functional metrics of ALS disease progression (FVC %predict, ALSFRS-R score, etc.) are better predictors of when Bi-PAP should be initiated and of its overall survival benefit compared to temporal metrics of disease progression likely due to highly heterogeneous disease courses and durations in the ALS population.

## Author contributions

NK data acquisition, statistical analysis, interpretation, critical review of the manuscript. GC data acquisition, statistical analysis, critical review of the manuscript. BW conception of study design, data acquisition. CM conception of study design, data acquisition, interpretation of results, drafting of the manuscript.

### Conflict of interest statement

The authors declare that the research was conducted in the absence of any commercial or financial relationships that could be construed as a potential conflict of interest.
